# The prevalence and correlates of social phobia among undergraduate health science students in Gondar, Gondar Ethiopia

**DOI:** 10.1186/s13104-019-4482-y

**Published:** 2019-07-19

**Authors:** Getachew Tesfaw Desalegn, Wondale Getinet, Getnet Tadie

**Affiliations:** Department of Psychiatry, College of Medicine and Health Sciences, University of Gondar, Gondar, Ethiopia

**Keywords:** Social phobia, Undergraduate university students, Prevalence

## Abstract

**Objective:**

Social phobia is highly prevalent among university students. The lowest and highest point prevalence of social phobia among undergraduate university students was estimated at 7.8% and 80%, respectively. However, research into social phobia and associated factors among undergraduate university students in low and middle-income countries has been limited. Therefore, this study aimed to assess social phobia and associated factors among university students in Ethiopia to contribute an attempt to ensure optimal care for students.

**Result:**

A total of 503 participants were interviewed with a response rate of 100%. The mean age of the respondents was 22.17 (± 10) years. The prevalence of social phobia symptoms among students was found to be 31.2% with (95% CI 27.3 to 35.6%). In the multivariable analysis, poor social support (AOR = 2.8, 95% CI 1.40, 5.60), female sex (AOR = 2.3; 95% CI 1.50, 3.60), 1st-year students (AOR = 5.5; 95% CI 1.80, 17.20), and coming from a rural residence (AOR = 1.6; 95% CI 1.00, 2.40) were factors significantly associated with social phobia symptoms.

**Electronic supplementary material:**

The online version of this article (10.1186/s13104-019-4482-y) contains supplementary material, which is available to authorized users.

## Introduction

Social phobia (SP) is the fear of social situations that involved interaction with others with its prevalence ranges from 3 to 13% in the general population [1]. Globally, the lifetime and current prevalence of social anxiety disorder was estimated at 4% and 1.3%, respectively [2]. Its onset started in late childhood and associated with new demands for social interaction, younger age, female sex, lower educational status, lower income, and performing in public [1, 3].

Social anxiety disorder was fearful in social gatherings, fear of public speaking, meeting new people, and avoidance of social situations [1–3]. Social fearful persons made bad images of their performance in social situations [4].

Social phobia was associated with problems within the siblings and the family [5]. The most common prevalence of social fear among the people was public speaking, and associated with female gender, low educational performance, psychiatric medication use, and absence of social support which led to low self-esteem, more distorted body image, and difficulty to interact with a social environment [4, 6–8].

Social phobia was a high prevalence among high school, college, and university students [9–12]. Two studies were done among undergraduate university students: the point prevalence of social phobia estimated at 7.8% and 80%, respectively [9, 13]. Different studies revealed that the major source of SP among university students was; exam, presentation, language, parental anger, criticism in front of others, exaggerated protection, maltreatment, and family provocation [12, 14]. Contributing factors for SP among students were a problem with the peers, roommates, feel that campus environment uncomfortable for study, racial diversity, and too many classmates were making study difficult [4].

Why students feared situations to diagnosis SP were giving talks in front of the audience and trying to make someone’s intimate romantic relationship [15, 16] and different studies reported risk factors of social phobia were; female sex, poor academic performances, psychoactive medication use, poor social support, freshmen, and spending more time thinking about face book [9, 17, 18].

The impact of social phobia among students decreased educational performance, dependence to take alcohol, avoid oral presentations, weak performance at clinical examinations, and develop depressive symptoms [13, 19].

Even though social anxiety disorder/social phobia has a high prevalence among university students globally including Ethiopia, little attention is given to its diagnosis and treatment. To the best of our knowledge, there has been no published study on social anxiety symptoms and associated factors among university students in Ethiopia. This study, therefore, aimed to investigate the prevalence and associated factors of social phobia symptoms among undergraduate students with a view to informing the development of interventions.

## Main text

### Methods

An institution based cross-sectional study was conducted at the University of Gondar from April to May 2018, Gondar Ethiopia.

Regular undergraduate students at the University of Gondar College of Medicine and Health Sciences were included in the sample and excluded critically ill students.

The sample size was determined by using the single population proportion formula involving the use of Epi-info version 7 with a 95% CI, a 4% margin of error, and a social phobia of 27.5% from previous study conducted among high school adolescents in Ethiopia [20]. Assuming a 5% non-response rate, 503 students were recruited randomly by using the simple random sampling technique. The total number of students in the college with their identification number taken from the UoG CMHS registrar office; then the required sample was selected through lottery method. The lists of dormitory students took from the UoG CMHS Student’s union dormitory affairs.

Data were collected using a pre-tested self-administered questionnaire, which contained socio-demographic factors, social support, clinical factors, and substance use factors. Social support was collected by the Oslo 3-item social support scale, which had a 3-item questionnaire commonly used to assess social support and used in several studies. The sum score scale ranges from 3 to 14, and had three broad categories: “Poor support” 3–8, “moderate support” 9–11, and “strong support” 12–14 [21]. Social phobia was measured by using 17 items social phobia inventory (SPI) scale with cut-off point’s ≥ 21. Its score ranges from 0 to 68, which was rated from 0 (not at all) to 4 (extremely) [22]. Social phobia inventory scale validated in different countries among adults and adolescents [23, 24].

Data were entered into Epi-info 7 software after checking for completeness and imported to SPSS version 21 for analysis. Univariate analysis was done to see the association of each independent variable with the outcome variable. Those variables a P-value less than 0.2 were entered into the multivariate logistic regression model to identify the effect of each independent variable with the outcome variables. The strength of the association evaluated by the adjusted odds ratio with a 95% CI, and less than 0.05 P-values were considered statistically significant.

### Results

#### Socio-demographic characteristics

A total of 503 students was included in the study with a response rate of 100%. The mean age of the respondents was 22.17 (± 10) years. Out of the participants, 362 (72%) were male, 472 (93.8%) were single, 289 (57.5%) were coming from the rural residence, and over two-fifth (43.3%) were between the ages of 18 and 21 years. Among the respondents, 185 (36.8%) were 3rd-year students and their grade scored between a range of 2.75 and 3.5 (Table 1).

**Table 1:** Distribution of students by socio-demographic factors at UoG, CMHS, in 2018 (N = 503)

Variables	Categories	Frequency	Percent
Sex	Male	362	72
	Female	141	28
Age	18–21	218	43.3
	22–24	214	42.5
	25 and above	71	14.2
Ethnicity	Amhara	348	69.2
	Oromo	70	13.9
	Tigray	39	7.8
	SNSS	23	4.6
	Others^a^	23	4.6
Religions	Orthodox	391	77.7
	Protestant	58	11.6
	Muslim	54	10.7
Marital status	Single	472	93.8
	Married	31	6.2
Residence	Urban	289	57.5
	Rural	214	42.5
Year of study	1st	69	13.7
	2nd	119	23.7
	3rd	185	36.8
	4th	103	20.5
	5th	27	5.4
Grade result	Below 2.00	11	2.2
	2.00–2.75	71	14.1
	2.75–3.5	249	49.5
	Above 3.5	172	34.2

^a^Somali and Afar

#### Clinical, social, and substance characteristics

A small number, 13 (2.6%) of the participants had history of mental illness, 84 (16.7%) had a chronic physical illness, and about 3.2% had family history of mental illness. Of the participants, almost two in five (43.3%) students had moderate social support and nearly two in five (41.4%) had poor social support. Regarding the current use of the substance: over two-thirds (43.7%) of the students were drinking alcohol and 56 (11.1%) were taking khat at the movement (Additional file 1).

#### Prevalence of social phobia

**Fig. 1:**
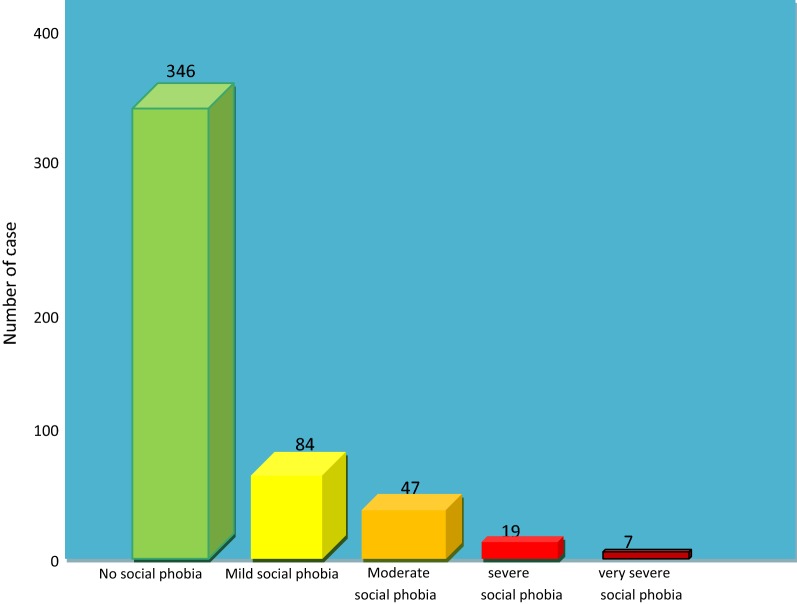
Bar chart showing that the distribution SPI score for students at the University of Gondar, Northwest Ethiopia in, 2018 (N = 503)

The 17-items of social phobia inventory were summed and the single variable was generated. The new variable ranges from 0 to 68 in absolute value. A total of 84 (16.7%) students had mild social phobia (scored about 21 to 30) and 47 (9.3%) of the students had a moderate social phobia (scored 31 to 40). A small number, 19 (3.8%) and 7 (1.39%) of the students had severe and very severe social phobia, respectively (Fig. 1). We further categorized social phobia into two levels (no social phobia and social phobia). This study showed that the prevalence of social phobia symptoms among participants was 31.2% with (95%, CI 27.3, 35.6%) (Additional file 2).

#### Factors associated with social phobia

Among all covariates, female sex, students studying in the 1st year, family history of mental illness, and poor social support had less than 0.2 a P-value in the univariate logistic regression and were considered as the multiple logistic regression models.

In the multivariable analysis suggested that the odds of social phobia, increased by 2.8 times (95% CI 1.40, 5.60) for students had poor social support compared to students had good social support. Female students were about 2.3 times (95%, CI 1.50, 3.60) more likely risk of social phobia compared to counterparts. Students studying in the 1st year were 5.5 times (95%, CI 1.80, 17.20) more likely to develop social phobia compared to counterparts. Similarly, the risk of social phobia for students whose residence from the rural areas increased by 1.6 times (95%, CI 1.00, 2.40) compared to students whose residence from the urban areas (Table 2).

**Table 2:** Factors associated with social phobia among students in the University of Gondar, Northwest Ethiopia in, 2018 (N = 503)

Variables	Categories	Social phobia		COR (CI 95%)	AOR (CI 95%)
		No	Yes		
Sex	Male	263	99	1	1
	Female	83	58	1.86 (1.23, 2.80)	*2.3* (*1.5*, *3.60*)**
Years of study	1st	38	34	3.94 (1.34, 11.54)	*5.5* (*1.8*, *17.20*)*
	2nd	73	43	2.59 (0.92, 7.34)	3.7 (1.20, 11.10)
	3rd	137	48	1.54 (0.55, 4.30)	2.1 (0.70, 6.20)
	4th	76	27	1.56 (0.54, 4.54)	1.8 (0.60, 5.50)
	5th	22	5	1	1
Residence	Rural	136	78	1.52 (1.04, 2.04)	*1.6* (*1.00*, *2.40*)*
	Urban	210	79	1	1
A family history of mental illness	Yes	7	9	1	1
	No	339	148	2.94 (1.08, 8.06)	2.7 (0.90, 8.10)
Social support	Poor	138	91	2.84 (1.5, 5.5)	*2.8* (*1.40, 5.60*)**
	Moderate	152	53	1.5 (0.8, 3.0)	1.5 (0.70, 3.00)
	Strong	56	13	1	1

P-value for Hosmer and Lemeshew test = 0.51 social phobia

* Significant association (* P-value < 0.05 and ** P-value < 0.01), n = sample size

### Discussion

In this study, the prevalence of social phobia and possible association with various factors was assessed. The results of the present study revealed that a remarkable proportion of students had social phobia. The prevalence of social phobia among students was found to be 31.2%.

Regarding prevalence, our result is consistent with those of other studies carried out in Ethiopia, Nigeria, India, and Australia the prevalence was estimated at 27.5%, 31.1%, 28.6%, and 30%, respectively [20, 25–27].

On the other hand, the current study finding was higher than those of other studies done in two areas of Saudi Arabia, Canada, Iran, and India, the prevalence was estimated at 14.1%, 16.3%, 7.9%, 17.2%, and 7.8%, respectively [5, 7, 9, 11, 28]. The variations might be the distinctions in sample sizes, measurement tools, rating scales, gender differences, and the socio-cultural contrast between Ethiopia and the other countries. In two areas of Saudi Arabia, the sample size was higher than in our study, while the measurement tool was the same [5, 28]. Besides the above differences, in two areas of Saudi Arabia took male and female students in their studies respectively [6, 27]. In Canada, Iran, and India, the diagnostic interview schedule III, Leibowitz questionnaires, and social interaction anxiety scale tools were used to assess the social phobia among university students, respectively.

However, our result was lower than those of other studies conducted in Saudi, India, Iran, two areas of Iraq and reported 60%, 46%, 78.9%, 58.5%, 80%, and 55.7%, respectively [12, 13, 19, 29, 30]. The discrepancy might be the sample size alterations and assessment tool differences. In Saudi, the study conducted among medical students and tested by using social phobia scale which differed from our assessment tool [19], in India, the study participants were only medical students but in our study all health science students included [30], in two areas of Iraq, college students and nursing students included in their studies respectively [13, 29].

Regarding associated factors, female sex was 2.3 times more likely at risk of increasing social phobia compared to male students. This study supported by those of other studies, females are not equally participated in all activities because of cultural influence when compared to male in Ethiopia [20], and in Iran, female students had highly prevalence of social anxiety disorder compared to male students [11]. The rate of specific phobias in women was double those of men [1]. The prevalence of social phobia near to double in female students compared to male students and the difference might be neglectful parenting styles and authoritarian difference between female and male students. Cultural and biological factors that may underlie sex differences in anxiety disorders [31, 32]. Social phobia has been faced comparatively high in female students compared to male students in our culture; males dominated and received special care from their parents. This is the major factors which affect the psychology of females and led to social anxiety symptoms and as a result, females have felt uncomfortable in social gatherings.

Poor social support was 2.8 times more likely to develop social phobia compared to good support this is comparable with the study done in Ethiopia [20], in Saudi Arabia, female students who had low income were exposed to social anxiety disorder [27], in India, medical students came from low socioeconomic class were a high risk of social phobia during their education [16], and school-age adolescents from urban residence had insufficient income families were more risk of social phobia [33].

Students studying in the 1st year were 5.5 times had social phobia compared to 5th-year students. This study was supported by other study was done in Indian medical college students compared to 2nd-year students but in our study the reference took from 5th-year students because most of the studies had low prevalent of social phobia in the last years of their study [17]. The 1st and 2nd-year students were highly risky for social phobia, the reason might be the University settings where they forced to live for away from their parents for the first time and expose for new environmental stressors including social situation [1, 34].

In Turkish, the 1st and 2nd-year university students had higher anxiety stress scores than other students [35]. Stress and environmental factors play a role in interpersonal stressors and thus can contribute to the development of social anxiety and differences in background, appearance, language, social and emotional development, all can affect whether or not a student fits in the university [36].

Finally, students coming from the rural areas were 1.6 times the risk of increasing social phobia compared to urban areas. Which was supported the studies done in India [14] and residence of students from the rural area in India medical college students developed social phobias which were consistency to our study [17]. Similar studies in Egypt, the prevalence of social phobia among male students were higher in urban areas but among female students were higher in rural areas [32] and the magnitude of social phobia was higher among rural areas’ students than urban and suburban students [37]. In Benin, the University of Parakou (UP) the impact of social phobia on academic performance among students living in rural areas were more risky to social phobia than those living in urban areas which means the prevalence of social phobia depends on the environment [34]. In Taiwan, rural adolescents were highly vulnerable to specific phobias compared to urban residences [38]. Another study conducted in India, risk factors of social anxiety in medical students were no significant difference between rural and urban residence [39]. The people living in rural areas were higher physical symptoms compared to those living in rural areas. The people belonging to urban areas had higher harm avoidance compared to those living in rural areas [36].

### Conclusion and recommendations

In this study, the overall magnitude of social phobia was found to be 31.2%. Female sex, poor social support, students studying in the 1st year, and rural residence were explanatory variables significantly associated with social phobia. The ministry of education and the University of Gondar better to develop guidelines to solve the aforementioned factors. Further research on risk factors for social phobia should be conducted to strengthen and broaden these findings.

## Limitations

A cross-sectional design cannot permit conclusions for some variables, for example, to decide whether social phobia symptoms are risks for or consequence. This finding is likely only to hint at the complex interactions between social phobia and explanatory variables (risk factors). The survey samples were a small number of students, the research work provided a summary of survey results. Another most important limitation of this study is the fact that the SPI scale was not validated in Ethiopia although it is widely used as a screening tool for social phobia in other countries. Further research should be considered on risk factors for social phobia to strengthen and broaden our results.

## Additional files

**Additional file 1.** Distribution of clinical, social, and substance characteristics of students at UoG, CMHS in, 2018 (n = 503).

**Additional file 2.** Pie chart distribution of social phobia among students in the University of Gondar, Northwest Ethiopia in, 2018 (N = 503).

## Data Availability

No additional file is available for this study; all the data are included in the manuscript

## Abbreviations

CBT: cognitive behavioral therapy
CMHS: College of Medicine and Health Science
DSM-V: Diagnostic and Statistical Manual of Mental Disorders, 5th edition
NCS-R: National Co-morbidity Survey Revised
PTSD: post traumatic stress disorder
SPIN: social phobia inventory scale
SPS: social phobia scale
UoG: University of Gondar
WHO: World Health Organization
