# Supplementary material for: The prevalence and correlates of social phobia among undergraduate health science students in Gondar, Gondar Ethiopia
Source: BMC Res Notes. 2019 Jul 19;12:438. doi: 10.1186/s13104-019-4482-y (PMC6642571; doi:10.1186/s13104-019-4482-y)
Supplement: Supplementary file 1 — Additional file 1. Distribution of clinical, social, and substance characteristics of students at UoG, CMHS in, 2018 (n = 503). [file 13104_2019_4482_MOESM1_ESM.docx]

Additional 1. Distribution of clinical, social, and substance characteristics of students at UoG, CMHS in, 2018 (n=503)

| Variables | Categories | **Frequency** | **Percent** |
| --- | --- | --- | --- |
| Social support | Poor | 229 | 41.4 |
|  | Moderate | 205 | 43.3 |
|  | Good | 69 | 15.1 |
| Known past psychiatric history | No | 490 | 97.4 |
|  | Yes | 13 | 2.6 |
| Known past medical history | No | 419 | 83.3 |
|  | Yes | 84 | 16.7 |
| A family known psychiatric history | No | 487 | 96.8 |
|  | Yes | 16 | 3.2 |
| Lifetime prevalence of substances | | | |
| Tobacco | No | 403 | 92.0 |
|  | Yes | 40 | 8.0 |
| Alcohol | No | 249 | 49.5 |
|  | Yes | 254 | 50.5 |
| Khat | No | 435 | 86.5 |
|  | Yes | 68 | 13.5 |
| Cannabis | No | 409 | 97.4 |
|  | Yes | 13 | 2.6 |
| The current prevalence of substance | | | |
| Tobacco: | No | 479 | 95.2 |
|  | Yes | 24 | 4.8 |
| Alcohol | No | 283 | 56.3 |
|  | Yes | 220 | 43.7 |
| Khat | No | 447 | 88.9 |
|  | Yes | 56 | 11.1 |
| Cannabis | No | 490 | 97.4 |
|  | Yes | 13 | 2.6 |
