# Supplementary material for: The prevalence and correlates of social phobia among undergraduate health science students in Gondar, Gondar Ethiopia
Source: BMC Res Notes. 2019 Jul 19;12:438. doi: 10.1186/s13104-019-4482-y (PMC6642571; doi:10.1186/s13104-019-4482-y)
Supplement: Supplementary file 2 — Additional file 2. Pie chart distribution of social phobia among students in the University of Gondar, Northwest Ethiopia in, 2018 (N = 503). [file 13104_2019_4482_MOESM2_ESM.docx]

No social phobia

Social phobia

Additional 2. Pie chart distribution of social phobia among students at the University of Gondar, Northwest Ethiopia in, 2018 (N=503).
